# The Shape of Ecosystem Management to Come: Anticipating Risks and Fostering Resilience

**DOI:** 10.1093/biosci/biu172

**Published:** 2014-11-05

**Authors:** Rupert Seidl

**Affiliations:** Rupert Seidl is assistant professor at the Institute of Silviculture, in the Department of Forest and Soil Sciences at the University of Natural Resources and Life Sciences (BOKU), in Vienna, Austria. E-mail: rupert.seidl@boku.ac.at

**Keywords:** risk, resilience, global change, ecosystem stewardship, sustainable management

## Abstract

Global change is increasingly challenging the sustainable provisioning of ecosystem services to society. Addressing future uncertainty and risk has therefore become a central problem of ecosystem management. With risk management and resilience-based stewardship, two contrasting approaches have been proposed to address this issue. Whereas one is concentrated on anticipating and mitigating risks, the other is focused on fostering the ability to absorb perturbations and maintain desired properties. While they have hitherto been discussed largely separately in the literature, I here propose a unifying framework of anticipating risks and fostering resilience in ecosystem management. Anticipatory action is advocated when the predictability of risk is high and sufficient knowledge to address it is available. Conversely, in situations in which predictability and knowledge are limited, resilience-based measures are paramount. I conclude that, by adopting a purposeful combination of insights from risk and resilience research, we can make ecosystem services provisioning more robust to future uncertainty and change.

**Global ecosystems support human well-being through** the provisioning of a wide range of ecosystem services (MA 2005). Providing food, freshwater, and fuel, they supply essential resources for human life on the planet. Ecosystems also deliver tangible benefits for society through regulating the environment—for example, by mitigating the processes of soil erosion and regulating the climate system. In addition, they provide services of cultural value, benefiting people through spiritual enrichment, cognitive development, reflection, and recreation. The backbone of a sustainable provisioning of these various services is formed by supporting services, such as primary production and nutrient cycling (MA 2005). The central goal of ecosystem management is to enable the sustainable provisioning of these ecosystem services to society while maintaining the integrity and diversity of ecosystems (Christensen et al. 1996). However, despite a growing scientific foundation for proper ecosystem management, the conditions of ecosystem services have largely declined in recent decades (Carpenter et al. 2009). Factors such as climate change, land degradation, biodiversity loss, and invasive alien species are increasingly challenging ecosystem services provisioning (Schröter et al. 2005), and uncertainty about their future development strongly complicates decisionmaking for ecosystem managers.

A primary approach for dealing with uncertainty in ecosystem management is adaptive management, in which management is conceived as a continuous cycle of planning, acting, monitoring, and evaluating measures (Westgate et al. 2013). Iterations through this cycle allow goals to be revised periodically, enabling the accommodation of changing societal demands for ecosystem services. It also allows periodic adjustments to changing environmental conditions and is therefore a primary approach to addressing climatic changes in ecosystem management. In adaptive management, it is recognized that knowledge about the future is always incomplete, and it takes its cues from monitoring and observations to assimilate new and emerging information into management decisionmaking. This cyclic updating works particularly well for gradual changes—that is, in situations in which the frequency of updating through the adaptive cycle is high relative to the rate of change. An important family of factors that pose uncertainty in ecosystem management are, however, low-probability, high-impact events (e.g., natural disturbances, climatic extremes, and ecological regime shifts). These are processes that substantially affect management and its objectives; however, they are relatively rare in their occurrence, and there are usually little to no *a priori* cues that would allow timely adaptation. In many instances, it will therefore be already too late to adapt to such events once their impacts become observable within the frame of the adaptive cycle. Because this is a general characteristic of many risk processes, ecosystem management has recently turned to acknowledging risks more explicitly in its decisionmaking.

Risk management approaches (*sensu* ISO 2009) are increasingly adopted into the ecosystem management practice in order to address pressures and threats to ecosystem services provisioning. Please note that I refer here to risks with regard to the objectives of ecosystem management and not to the risk of unintended consequences of human activities on ecosystems, which are studied by means of ecological risk assessments (see, e.g., Chen et al. 2013). These efforts to quantify and manage risks in ecosystem stewardship *inter alia* draw on the extensive experience of risk management in other areas (ISO 2009, Purdy 2010). Their overall aim is to anticipate the manifestation of risks and mitigate their impact. However, another paradigm to address change and deal with low-probability, high-impact events in ecosystem stewardship has also emerged recently. *Resilience*—that is, the capacity of a system to absorb perturbations and retain structures and processes—is now widely perceived not only as an important property of ecosystems but also as a model to manage coupled human and natural systems (Liu et al. 2007). Resilience-based stewardship is increasingly advocated as a means to cope with uncertainties and risks in ecosystem management (Biggs et al. 2012). Conspicuously, the concepts of risk and resilience—emerging from different communities and contexts—have been discussed largely separately in the ecosystem management literature to date (see the supplemental material).

In order to amend the toolbox of adaptive management with regard to an approach for dealing with low-probability, high-impact events, I propose here a unifying framework of anticipatory risk management and resilience-based stewardship in ecosystem management. This framework is based on the explicit consideration of uncertainties with regard to the predictability of risk, as well as the available knowledge of how to address it, an idea first proposed by Wildavsky (1988) that has, however, not yet been discussed in and adopted to the stewardship of biological systems to date (but see de Bruijne et al. 2010 and Howell 2013 for applications in other fields and contexts). I start by briefly summarizing the current approaches of risk management and resilience-based stewardship in ecosystem management. Subsequently, I develop a framework of how to jointly incorporate them into management decisionmaking and present examples of how this framework can be applied in the management of different ecosystems and objectives. I end by highlighting some of the remaining obstacles to comprehensively addressing uncertainty and risk in ecosystem management.

## Anticipatory risk management

The effect of uncertainty on objectives is referred to as *risk* (ISO 2009). Despite the large number of factors introducing uncertainty in ecosystem management and their potentially high impact on ecosystem services, the most common way of dealing with risk in traditional approaches to manage ecosystems was probably ignorance (Puettmann et al. 2009, Woods and Coates 2013). Extreme events and disturbances were often accepted as *force majeur* and, therefore, were not explicit considered in management decisionmaking, despite their potential to considerably influence management outcomes (Francis et al. 2007, Howden et al. 2007). Ignoring risk, however, has been shown to lead to suboptimal management decisions and can result in a considerable bias in the assessments of future trajectories of ecosystem services (Kurz et al. 2008, Woods and Coates 2013).

Consequently, risk management has gained increasing attention in the ecosystem management community, and a variety of risk management approaches have been presented in recent years. In general, the risk management process involves establishing the context of the risk management problem, assessing the risk, and treating the risk (ISO 2009). The central element of risk assessment usually consists of risk identification, risk analysis, and risk evaluation (Purdy 2010). Risk management frameworks have been applied to a variety of risk factors, including the risk from natural disturbances such as wildfires (Fairbrother and Turnley 2005). The risks from climate change are also increasingly addressed using risk management frameworks (Howden et al. 2007). Although many early approaches were focused on a single risk factor, recent efforts have been aimed at developing frameworks to address compounding risks. Jactel and colleagues (2012), for instance, used multicriteria analysis techniques to integrate multiple risk factors in the context of managing natural disturbances and invasive species in forest ecosystems.

A common theme in many of these risk assessment approaches is their anticipatory nature. Their aim (implicitly or explicitly stated) is to identify specific risks and implement measures to mitigate them. The options typically considered to treat risks are to avoid them, to remove the risk source, to reduce the likelihood or consequences, and to share and distribute the risk (Purdy 2010). Examples in ecosystem management include aiming to achieve more stable and less risk prone structures—for example, through adapting the species composition, altering management frequency and intensity, and using pest control measures. In the same context, insurance models to share risk are increasingly considered to counter growing environmental risks (Mills 2007).

## Resilience-based stewardship of ecosystems

With a focus on controlling uncertainties and their consequences, risk management concepts are firmly rooted in the ideas and approaches of the engineering sciences. Recently, resilience has emerged as a new paradigm of ecosystem stewardship in the face of change and uncertainty (Biggs et al. 2012), originating from ecological ideas on disturbance and recovery (Holling 1973). The concept of resilience has received considerable attention lately and has been adapted to a variety of issues, ranging from ecosystem management and disaster response to global governance of the biosphere. Consequently, a large number of different definitions of resilience exist today. For the purpose of this contribution, I define *resilience* as the capacity of a system to absorb perturbations and to retain essential structures and processes (i.e., here, the sustainable provisioning of ecosystem services; Carpenter et al. 2001). For a comprehensive review of resilience in the context of ecosystem management, I refer to Biggs and colleagues (2012). In the following, I will briefly summarize selected aspects of resilience thinking and highlight how they differ from ideas of risk management.

Resilience-based management acknowledges that resource management decisions are always made in an environment of uncertainty. Change and uncertainty are therefore viewed as inherent to the system, making them a central aspect of management considerations. As such, many aspects of resilience thinking are not focused primarily on the *before* state (i.e., on preventing the manifestation or impact of a risk, as is the case with risk management) but at least equally on the *after* state (i.e., on the mechanisms of coping with perturbations and on bouncing back through trajectories of recovery). Whereas a central goal of anticipatory risk management approaches is to maintain and increase stability via reducing risk factors and their impacts, resilience theory suggests that overly stable systems become increasingly brittle and prone to perturbations. Resilience thinking therefore acknowledges that small disturbances can reduce the vulnerability to large perturbations rather than recommending the prevention of disturbances altogether (Biggs et al. 2012).

Another central theme of resilience research is adaptiveness. Particularly in the context of how to deal with climate risks, this aspect has recently received increasing attention in ecosystem management (Howden et al. 2007, Lindner et al. 2010). In this regard, the resilience concept has helped to mainstream a complex adaptive systems perspective into ecosystem management (Francis et al. 2007, Puettmann et al. 2009), highlighting the importance of self-organized behavior of adaptive agents in coping with perturbations. Furthermore, the central role of diversity in dealing with uncertainty is a keystone element of resilience-based management (Mori et al. 2013), and diversification (with regard to ecosystem structure and composition) has been shown to be an important strategy to deal with, for example, climate risks (Seidl et al. 2011a). Another important contribution of resilience research to issues of ecosystem management relates to a growing understanding of the response of ecosystems to perturbations. Research in this field has, for instance, highlighted that ecosystems can respond strongly and nonlinearly to pressures and can flip into an alternative state (threshold behavior) if the resilience of the system is exceeded (Carpenter et al. 2001).

## A unifying framework of anticipating risks and fostering resilience

The main motivation for considering risk and resilience in ecosystem management is uncertainty, a factor that is omnipresent in all management decisionmaking.

### Uncertainty as the problem

An important and long-recognized source of uncertainty in ecosystem management is the market and its fluctuations. Considering the long production periods in forestry, for instance, predictions of market developments are highly uncertain and exert an economic risk on forest management decisionmaking (Pukkala and Kellomäki 2012). Put more generally, the future societal preferences and valuations of ecosystem services (reflected in market prices in the case of services with a well-established monetary market) are uncertain. As a result of societal changes, particular ecosystem services can rapidly gain in importance, sometimes at the cost of others. The developments in the Pacific Northwest of the United States in the 1990s can serve as an example here, with timber production from federal forest land collapsing to approximately 10% of the level of the previous decade as a result of increasing conservation concerns by vocal parts of society (Thomas et al. 2006). Furthermore, newly emerging management objectives provide evidence of changing societal preferences for ecosystem services: Managing ecosystems to lower the atmospheric greenhouse gas content, which is currently an important issue of ecosystem management (e.g., in agriculture and forestry), was not on the agenda of managers only a couple of decades ago. These two examples document that ecosystem management has to deal with considerable uncertainty regarding future societal demands.

Another group of factors introducing uncertainty relates to changes in the environment. Climate risks, for instance, are expected to increase drastically in the coming decades (IPCC 2013). The risks related to environmental changes include a loss of productivity and economic potential, as well as increased levels of fluctuation and natural disturbance. Climatic changes therefore have the potential to distinctly alter the provisioning of ecosystem services (Schröter et al. 2005). Many traditional ecosystem management paradigms have been developed under the assumption of stable environmental conditions (Woods and Coates 2013), and the mainstreaming of a changing environment into operational management decisionmaking has proven to be slow (see Blennow and Persson 2009). Whereas climatic risks are a relatively new addition to the risk portfolio, natural disturbances, pests, and diseases are risk factors that are more frequently considered in ecosystem management. They have long been recognized as “stochastic,” “unpredictable” elements in the management of ecosystems, with the potential to cause substantial loss in ecosystem services. In this context, it is important to note that individual sources of uncertainty rarely operate in separation but are usually connected with or conditional on other factors. Climate change is, for instance, expected to further amplify disturbance processes and the occurrence of pests and diseases (Dale et al. 2001), which underlines that the interconnectedness of uncertainty generally increases its magnitude and impact. But not only can environmental sources of uncertainty be connected, but the uncertainties of social and ecological spheres can also interact: An example is invasive alien species that are facilitated by human activity and trade, which increasingly cause problems for ecosystem management (Leung et al. 2012).

### Uncertainty as the solution

Global change considerably increases uncertainties in ecosystem management (Young et al. 2006). Concurrently, the demand for ecosystem services increases, which aggravates the potential impacts of uncertainty on the goals of ecosystem management. A prime challenge for current ecosystem management is therefore to reduce uncertainties and to mitigate their potential negative impacts on ecosystem services. In view of a growing understanding of many risk factors, managers would act irresponsibly (both toward their employer and toward society at large) if they would not aim to anticipate risks and prevent (or at least mitigate) their negative impacts. Furthermore, in many instances, risk prevention is far more resource efficient than coping with impacts from the manifestation of risk. Anticipatory risk management therefore plays an important role in ecosystem management in a changing world. However, despite a long history of risk management, many risk factors have recently been gaining rather than losing importance. Moreover, with risks from disturbances and diseases, invasive alien species, and climate change being predicted to increase further in the near future, it is increasingly unlikely that anticipatory risk management will be able to fully prevent losses from such risk factors. Resilience-based ideas are therefore equally needed to cope with these impacts and to accommodate previously not considered uncertainties (“unknown unknowns”) in ecosystem management. Exclusively aiming to prevent known risks (and ignoring the possibility of unknown and unintended consequences) is equally shortsighted as solely fostering resilience (while accepting losses from impacts that could have been prevented).

**Figure 1.**
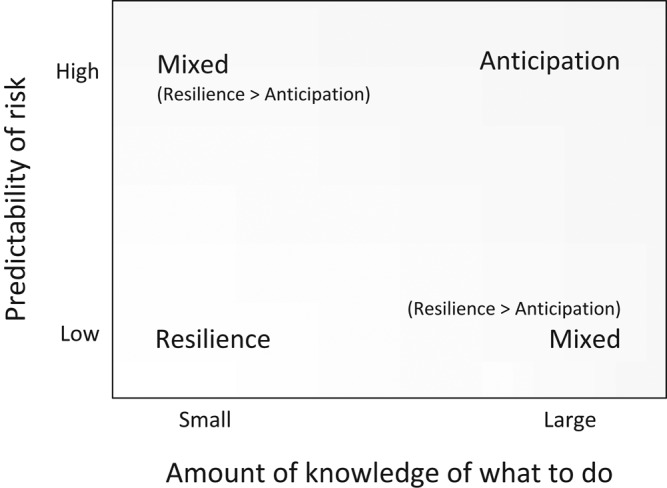
A framework for dealing with uncertainty and risk under different conditions of predictability and knowledge about effective measures. *Anticipation* here refers to measures aimed at reducing the risk or impact from a specific risk factor, whereas *resilience* refers to measures that foster the ability of the system to absorb perturbations and to maintain essential functions and services. *Mixed* refers to a combination of anticipatory and resilience-focused measures. Source: This image was adapted from Searching for Safety by Aaron Wildavsky. Reused with permission from Transaction Publishers. Copyright 1988.

Whether anticipatory or resilience-based management approaches are more promising should be considered at the level of specific management decisions and depends on the predictability of risk and the knowledge about effective measures of how to deal with it (Wildavsky 1988). Anticipation is effective in coping with known threats (e.g., the upper right quadrant in figure 1). It reduces the occurrence of specific perturbations or increases systems’ resistance to them. Anticipation becomes inefficient, however, when uncertainty about the risk factor increases or when the knowledge about how to address the risk is lacking, which is the domain of resilience-based measures. If effective measures to address a risk would be available but the risk is virtually unpredictable, a mix of anticipatory and resilience-focused approaches is commendable. The same applies when we know what to expect but do not know what to do about it. In cases in which a mixed strategy is advisable, it is prudent to give priority to resilience over anticipation, because the generic resources of resilience thinking (e.g., self-organization, response diversity, adaptive capacity) are more likely to enable management to respond to future surprises (Wildavsky 1988).

The predictability of and knowledge about responses are both context-specific—that is, there is no one general position along these two axes for the problem of addressing uncertainty and risk in ecosystem management. Considering the risk of climate change, for instance, it is virtually certain that climatic conditions are going to be different by the end of the twenty-first century from those of the recent past (IPCC 2013). This exerts a strong imperative to act anticipatorily and to adapt to these expected changes (Kolström et al. 2011). However, both the exact local manifestations of future climatic change and their effects on ecosystems remain uncertain. A good measure of resilience thinking is therefore required in any climate change adaptation strategy (Seidl and Lexer 2013).

In general, many institutions and guidelines for dealing with risk and uncertainty in ecosystem management are currently focused on anticipatory measures. These have the advantage that they are directional and take effect directly, and therefore result in clear and measurable results (at least with regard to their implementation, but not necessarily with regard to their desired effect on risk), which is important in institutional frameworks requiring formal control and accountability (e.g., in governmental agencies). Measures promoting resilience, however, frequently fall short on producing immediate, specific results. However, a sole focus on anticipatory command-and-­control ­management can create brittleness and can lead to the inability to react to unexpected future outcomes (Holling and Meffe 1996).

## Management examples

The following paragraphs give examples of how this framework of anticipating risks and fostering resilience can be applied in the management of different ecosystems and objectives.

### Forest ecosystem management

**Figure 2.**
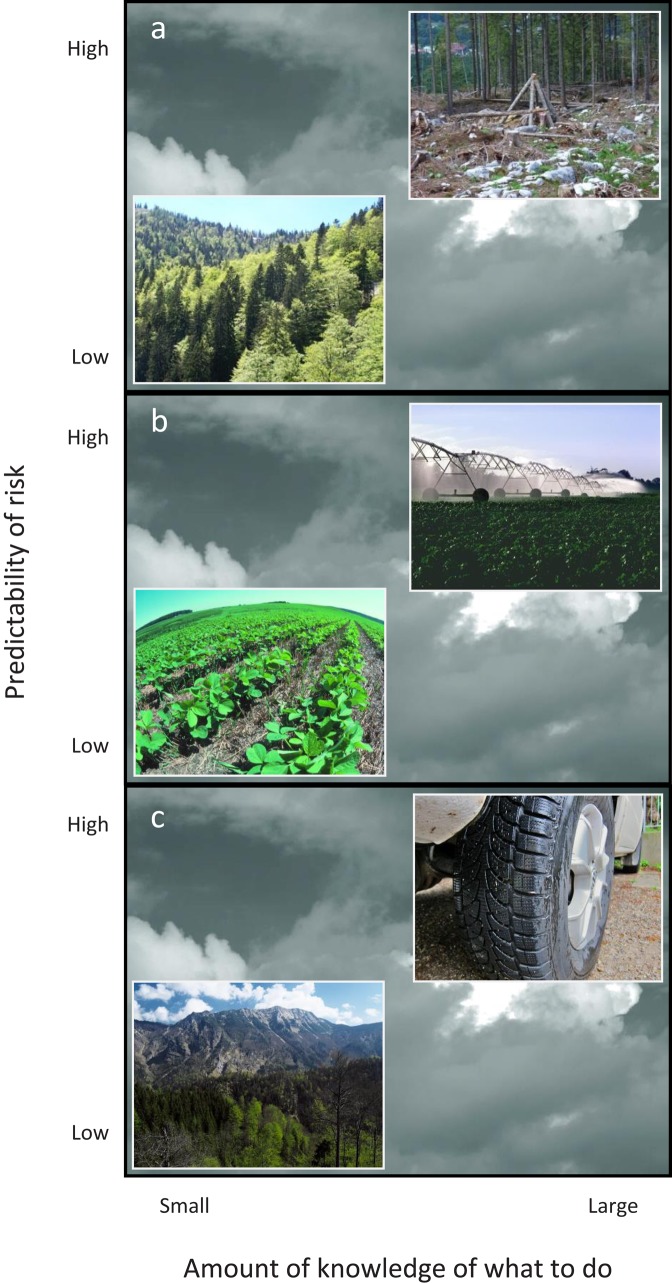
Anticipatory risk management measures (the upper right image in each panel) and measures fostering resilience (the lower left image in each panel) in the context of (a) disturbance management in forest ecosystems, (b) drought management in agroecosystems, and (c) invasive alien species management in conservation. (a) The risk of further attack from bark beetles at the edge of previous year's outbreak front is high, and ample knowledge of measures of how to dampen the further spread of an outbreak is available. Anticipatory risk management, shown here in the form of a beetle trap with a pheromone dispenser (the upper right image), is therefore a highly feasible management option. Considering larger spatial and longer temporal scales, however, the predictability of where and when a bark beetle outbreak will occur is low, making specific anticipatory measures impossible. At these scales, the resilience to such disturbance events can be increased, for example, by lowering the share of host species for aggressive bark beetle species and increasing the response diversity of the system (the lower left image). (b) In order to address the expected increase in drought risk in agriculture, irrigation systems can be extended and improved in order to sustain crop yield in areas that are expected to suffer particularly from future climate change (the upper right image). Considering the limited water resources for irrigation and the possible negative effects on soil erosion, as well as the fact that the precise prediction of the timing and location of future drought is impossible, soil and soil water (and therefore the resilience of agroecosystems) should be protected by, for example, applying no-till farming techniques (the lower left image). (c) Anticipatory measures to prevent the spread of invasive alien species into areas of particular interest for conservation include the inspection and cleaning of vehicles (particularly, the tires and undercarriage) before entering the area (the upper right image). The resilience to invasion by alien species can be increased by restoring natural systems, increasing the connectivity of the landscape, and setting aside sizeable and heterogeneous landscapes for conservation (the lower left image shows the recently extended Dürrenstein wilderness area in the northern Austrian Alps). Photographs: Rupert Seidl, USDA.

In long-lived ecosystems such as forests, the climatic changes expected for the future will unfold very rapidly relative to the century-long process of natural forest development and succession. Because this temporal mismatch limits the possibility of iterative adaptation through the adaptive cycle, the anticipation of predictable risks is of considerable importance in forest management. In anticipation of a warming world, it might therefore be advisable to adjust the tree species composition to more warm- and drought-tolerant species (through planting or assisted migration) in order to sustain important ecosystem services (Kolström et al. 2011, Seidl et al. 2011a). Furthermore, the thinning intensity could also be increased in order to reduce the competition between individuals and, therefore, to alleviate the aggravating resource situation (e.g., with regard to water) for the remaining trees (Magruder et al. 2013). However, many changes in forest ecosystems do not progress gradually but take place after disturbance breaks the connections within the system and frees resources. The expected increase in natural disturbances under climate change can therefore facilitate forest change. Although some anticipatory measures against intensifying disturbance regimes can be taken (e.g., increasing the stability of individual trees and forest stands), the precise occurrence and impact of a disturbance event remains unpredictable. It is therefore important to facilitate the resilience to such events—for example, by promoting diversity in species and structure (and, consequently, response diversity; Mori et al. 2013), in order to support the ecosystem in recovering its structures and functions after disturbance (see figure 2a). Here, it is important to focus not only on tree- or stand-level processes because landscape-scale elements such as disturbance legacies (i.e., biological remnants that are carried over from the predisturbance forest) can also significantly enhance resilience (Seidl et al. 2014). A shortening of rotation periods can serve as an anticipatory measure to reduce the risk from natural disturbances (because disturbance risk for many agents increases with stand age) and can also foster adaptation to changes—for example, through adaptation of the prevailing species composition in the subsequent generation. This anticipatory measure, however, also aptly illustrates the complexity of managing forests under uncertainty: Because many forest managers need to balance a multitude of ecosystem services provided by the area under their stewardship (a fact that distinguishes forest management from, e.g., agriculture), the trade-offs inherent in measures such as reducing the rotation period in response to increasing uncertainty (i.e., potential positive effects on timber production through less disturbance damage but also potential negative effects on carbon storage due to a younger forest demographic on the landscape) need to be explicitly considered. Furthermore, because the structural complexity associated with old forests have been linked to high levels of resilience, this example also highlights the inherent trade-offs between measures anticipating specific risks and those fostering resilience. A specific example of how to resolve these trade-offs when adapting forest management to climate change, using the uncertainty-based framework presented here, is given in the supplemental material.

### The management of agroecosystems

Feeding a growing world population is one of the biggest challenges for ecosystem management. Adding to this challenge is the considerable vulnerability of agricultural systems to a changing climate (Howden et al. 2007). Although primary productivity will benefit from the expected climatic changes in some regions of the world (e.g., in northern countries), the expected increasing drought frequency poses a considerable risk for agriculture. The shorter production cycles in agriculture (relative to the decadal- to century-scale changes in climate) generally allow for more iterations through the adaptive cycle and a faster adjustment to changing environmental conditions (a process that can already be observed; see e.g., Olesen et al. 2011). This—in theory—reduces the importance of anticipatory action. In practice, however, adaptation measures such as changing crop species or the timing of management operations (e.g., sowing, harvesting) also require the mainstreaming of new knowledge and techniques into farming practice, which could delay adaptation and could increase climate-related losses. Anticipating future climate risks such as more-frequent and severe droughts has the potential to make global food supply more robust with regard to expected future climate risks. Measures such as breeding programs for more drought-tolerant crop species and the optimization and extension of irrigation systems, for instance, can help to reduce the drought risk (Witcombe et al. 2008). Because evidence for increasing drought frequency and severity is mounting (IPCC 2013) and given the extensive knowledge of agricultural production in dry and drought-prone areas, anticipatory action to reduce drought risks in agriculture is increasingly warranted (cf. figure 1).

However, changes in the water cycle—both climate-change-related and due to an increasing use of irrigation—also hold the potential for less predictable and addressable threats to agriculture in the future. In many areas of the globe, the precipitation regime is expected to become more extreme with regard to both dry and wet tails of the distribution, and an increase in heavy rainfall events is projected for many areas of the globe (IPCC 2013). Such trends will intensify erosion and soil loss (Routschek et al. 2014) and can subsequently decrease agricultural production and hamper food security. Intensified irrigation could further amplify erosion and could change sedimentary dynamics (Stoate et al. 2009). These changes in the hydrological cycle therefore have the potential to deteriorate the fundamental base of any primary production. Because it is not possible, as it is with drought impacts, to alleviate heavy precipitation events through anticipatory measures, soil erosion is not reversible, and soil formation happens only on geological time scales, maintaining soils and their capacity is fundamental for the resilience of agroecosystems (see figure 2b). Fostering this capacity—for example, through promoting species that stabilize the soil, changes in tillage practices to conserve water and prevent soil erosion, and a more holistic management of water resources—will therefore become increasingly important in the future (Howden et al. 2007, Olesen et al. 2011). A balance between such resilience-centered measures and measures to anticipate risks and increase productivity can be found by locally assessing the respective uncertainties associated with risk factors such as drought and soil erosion (see figure 1).

### Conservation management

In addition to soils and their fertility, biological diversity is another key element of ecological resilience and forms the backbone of many crucial ecosystem functions and services (Cardinale et al. 2012). In addition to its intrinsic value, conserving biodiversity is a coarse filter approach to safeguarding the ability of ecosystems to adapt to changes. Conservation mangers around the globe therefore aim to preserve and foster biodiversity, both in managed landscapes (e.g., through specific contracts with land managers) and in dedicated protection zones (such as national parks and wilderness areas). As are many other ecosystem managers, conservationists are faced with considerable uncertainties and risks regarding their management objectives, among which climate change and invasive alien species are of increasing concern (Leung et al. 2012). Facilitated by globalization and intercontinental trade but occasionally also through active import and propagation by humans, an increasing number of plant and animal species are transplanted to regions and ecosystems in which they are not native. Once they are established, some of these alien species have the ability to aggressively invade natural ecosystems and to displace native and endemic species from their niches (Pyšek and Richardson 2010). These invasive alien species pose a considerable risk to conservation efforts. This risk is further increased by intensifying disturbance regimes under climate change and by the continued destruction and fragmentation of habitat (Marvier et al. 2004).

Although the problem of invasive alien species has increased in recent years, it is virtually unpredictable for an individual conservation manager which species (if any at all) will affect their focal ecosystem. Furthermore, because taking action against invasive aliens is ultimately dependent on their biology, anticipatory measures are rarely possible (cf. figure 1). Most efficient in addressing the risk from invasive aliens is therefore, in many cases, the facilitation of resilience, which may foster the capacity of the system or the focal organism of conservation to endure even if an invasive alien organism should enter the scene. To that end, native but regionally lost species could be reintroduced to fill respective open niches (Funk et al. 2008). Increasing the size of protected areas and fostering connectivity (e.g., through corridors) can also help endangered species endure in case of a future invasion (and, in addition, supports the autonomous adaptation of plant communities to a changing climate; see figure 2c). In some cases, however, concrete anticipatory measures addressing the risk from invasive alien species might also be advisable—for example, in order to prevent an already implanted species from spreading into a protected landscape. Focused eradication programs in the early stages of an invasion might also be able to stop an invasion. Often more efficient than eradication programs are anticipatory measures aimed at reducing the spread of invasive aliens—for example, through a focus on the pathways of spread and the potential vectors of invasive alien species (Pyšek and Richardson 2010).

Some species that are promoted as an anticipatory risk-mitigation strategy against future climate risks by some ecosystem managers are, at the same time, considered to be invasive alien species (and, therefore, risk factors) in the context of conservation. Douglas-fir (*Pseudotsuga menziesii* [Mirb.] Franco) is, for instance, promoted as a drought-tolerant, nonnative tree species in Europe in the context of climate change adaptation. However, concerns remain that a large-scale spread of the species could have negative impacts on biodiversity and would further distance managed forest ecosystems in the region from natural or close-to-natural conditions (Felton et al. 2013). These conflicting perspectives highlight the fact that considerations of risk and resilience should not be limited to one specific sector or land use but need to be negotiated holistically within a landscape.

## The challenges of addressing uncertainty and risk in ecosystem management

When making decisions about risk, I argue here that predictability and available knowledge should be used to balance between anticipatory and resilience-focused management measures. The examples given in the previous section underline the fact that the information for a comprehensive consideration of risk and uncertainty in operational management planning and decisionmaking is becoming increasingly available. However, considering that the mainstreaming of such ideas into operational ecosystem management is slow (Blennow and Persson 2009) compared with the pace of environmental and societal change, future research should particularly address the obstacles that currently hamper a comprehensive consideration of risk and resilience in ecosystem management. Four such major challenges are highlighted in the following section.

### Prediction

Ecosystem management is a forward-looking ­venture. Likewise, addressing risks in management entails making assumptions about the future. Predictions, whether they are implicit or explicit, are therefore a central part of ecosystem management in general and of addressing risks in particular. Especially in long-lived ecosystems such as forests, in which planning periods usually exceed multiple decades, robust knowledge about future trajectories of ecosystems and their services are in high demand. Because ecology is progressing toward a predictive science (Clark et al. 2001), these information needs are increasingly met by a growing science community. Such methodological advances increase the ability to quantitatively evaluate *what if* questions in the form of scenario analyses, the latter being a prime tool of uncertainty assessment (Walker et al. 2003). A prerequisite for such *what if* questions is that the processes pertaining to these questions are captured in the models used for prediction. A strictly empirical model, for instance, is parameterized on past data and, therefore, by design only contains information about factor levels and combinations that have been observed in the past. Future climate change, however, will likely bring about a no analogue future; that is, it will move the Earth system into uncharted territories outside the realm of past observations. When using models to make inquiries about the risk from future changes in the environment, it is therefore imperative to employ approaches that incorporate an understanding of the underlying processes affected by such changes (Evans 2012). Although our knowledge of ecosystem processes has been growing considerably in recent decades, many processes that pose risks in ecosystem management are still poorly understood. With regard to natural disturbances, for instance, the overwhelming majority of the modeling approaches presented in the last 15 years were descriptive, empirical models (Seidl et al. 2011b) documenting a still limited inferential potential under global change.

### Complexity

Although models and scenario analyses offer little help with anticipating unknown unknowns (we need to know the *if* beforehand in order to being able to ask *what if* questions), they support a systematic and quantitative analysis of the complexity of ecosystems. Ecological complexity relates to the diversity, nonlinearity, interconnectedness, and spatiotemporal heterogeneity of ecosystems. Ecosystems are increasingly recognized as complex adaptive systems, in which diverse agents interact with each other and their (heterogeneous) environment across a variety of hierarchical scales (Levin 1999). The bottom up, decentralized control through individual agents adapting to their environment leads to emergent phenomena—that is, behavior that cannot be observed at the level of individual components of the system. Recognizing and understanding such emergent behaviors is of crucial importance for addressing risks in ecosystem management, because it is at the core of a system's resilience to perturbations. Complexity therefore makes a key contribution to the ability of ecosystems to absorb manifest risks. However, accommodating this complexity in our predictions remains a considerable challenge. Addressing emergence in hierarchical systems, for instance, requires a multiscale approach to analysis and prediction: For example, the resistance of a forest to strong winds can be quantified at the level of individual trees. However, considerable additional information (e.g., on the distribution and spatial arrangement of trees) is required in order to estimate the risk to wind damage at the stand and landscape scale. Dynamically addressing such risks therefore requires scaling across multiple hierarchical levels (i.e., from leaves to landscapes), which, as is described in more detailed in the following section, poses a considerable methodological challenge (see Seidl et al. 2013). A further challenge related to complexity arises from the importance of interactions between processes and agents in complex adaptive systems. Identifying which interactions matter for answering the questions of risk management and accounting for them in predictions can be—at times—a daunting task.

### Scaling

**Figure 3.**
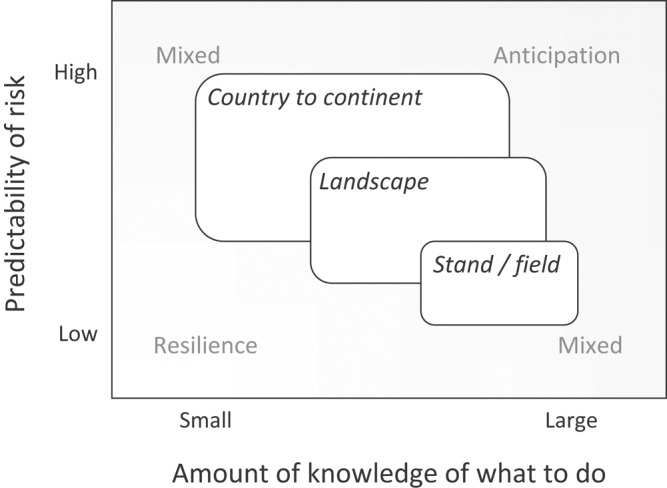
Stylized relationship of spatial scale to the predictability of risk and the knowledge of how to address it.

As outlined in the previous section, ecosystems are hierarchical multiscale systems, and scaling (i.e., a change in resolution or in extent with regard to the temporal or spatial representation of the system) is considered to be among the central problems in ecology. One aspect of particular relevance in the context of ecosystem management under uncertainty and risk is the observation that variability in ecosystems is conditional on the scale of observation (Wiens 1989). Consequently, predictability often increases when moving from individual cases to collections thereof (i.e., when scaling up). The same is true for the predictability of risks in general and for low-probability, high-impact events in particular (cf., the *y*-axis in figure 1), which generally increase with scale (see figure 3). At the landscape or country scale, it is virtually certain that climatic extremes or disturbance damage will occur on a fairly regular basis. At the scale of individual fields of crop or stands of trees, however, such events occur only infrequently, and although probabilities of occurrence can be estimated, the actual manifestation of an event remains unpredictable at this scale. Although the predictability of risk increases with scale, the knowledge of efficient means in responding to risk (i.e., the *x*-axis in figure 1) generally decreases with scale. Although we know quite well how to manage individual fields of agricultural crops or stands of trees to make them less prone to disturbance and disease, devising efficient risk-mitigation and response strategies at the landscape or even country scale is exceedingly difficult (not least because most of our understanding of risk factors comes from small- to medium-scale observations and experiments). Scale is therefore an important determinant in addressing uncertainty and risk in ecosystem management and should receive increasing attention in research and management (Seidl et al. 2013).

### Ecosystem–society interactions

Ecosystem management, risk, and resilience are not mere technical issues, and knowledge about the ecology of risk factors alone is not sufficient to holistically address them in ecosystem management. Recently, a coupled human and natural system perspective has emerged as a new paradigm for ecosystem management (Liu et al. 2007), which underlines the importance of the interactions between society and ecosystems for natural resource management. In the context of uncertainty and risk, one important issue in this regard is that what is perceived as a risk and how acceptable these risks are profoundly ­differs between individual actors of ecosystem management. For instance, risks that are familiar and that are thought to be under one's control are more accepted than are exotic risks perceived to be controlled by others (Fischhoff et al. 1981). In addition to the variability at the level of individual decisionmakers, institutions (e.g., government agencies, private enterprises) also differ in their levels of risk perception and risk aversion (Fairbrother and Turnley 2005). Many stakeholders of ecosystem management articulate risk-averse preferences, which is not always consistent with the actual decisions taken in operational management. Spatial configuration of, for example, ownership also influences risk perception and risk-mitigating behavior (Busby et al. 2012). Ultimately, preferences about objectives and about the level of change that is tolerated exert a strong influence on the assessment of the vulnerability to risk and the subsequent deduction of management responses (Seidl and Lexer 2013).

Another aspect that follows from adopting a coupled human and natural systems framework is that soundly predicting risks and knowing what to do about them is not sufficient to address them in management. Both trust (in information, institutions, individual actors) and the potential to implement measures (both technical and economic) are strongly dependent on the beliefs of the actors involved (Blennow and Persson 2009). Communication is therefore of paramount importance, and the way relevant information is framed influences the acceptance of management measures in the context of risk and uncertainty (Wilson et al. 2012). Managing risk factors such as disturbance is further complicated by the fact that they require a landscape scale perspective, and the cooperation of actors at large scales depends on people's beliefs and norms about reciprocity, as well as on the (perceived) benefits of interacting with others (Fischer and Charnley 2012).

## Outlook

Although the probabilities for many important risk factors in ecosystem management are still comparably low, their impact often has profound and long-lasting effects, because many ecosystems are slow in, rapid out systems (Körner 2003). In a typical old-growth forest, for instance, the carbon stored in the ecosystem (i.e., a measure for the stakes at risk) is one to two orders of magnitude larger than the annual net primary productivity (i.e., the main process of recovering carbon stocks). A disturbance event that occurs only once in several centuries can release large amounts of carbon into the atmosphere in a matter of weeks and can turn the system from a carbon sink into a carbon source for decades afterward (Kurz et al. 2008). Given the ongoing changes in the environment, the probabilities for such rapid out events are increasing (Lindner et al. 2010). The ecosystem management of the future will therefore have to be focused on more systematically addressing such risks from low-probability, high-impact events and must make (ecological as well as social) uncertainty a pivotal aspect for stewardship decisions. In extension of adaptive management, I here have outlined a strategy of how to address this increasingly central problem of ecosystem management, unifying approaches from anticipatory risk management and resilience thinking into a comprehensive framework to address uncertainty and risk.

Notwithstanding the growing relevance of risk, it is important that the desire for (individual) safety and stability must not lead to iatrogenic effects. The very nature of risk is that it is uncertain, and probability and magnitude are usually inversely related. Anticipatory risk reduction treatments can lower the probability of large events but might also directly and adversely affect the very ecosystem services they were designed to safeguard. Analyses of fuel treatments, for instance, show that although they reduce the risk for carbon loss from a subsequent large, high-severity fire, they—when the factor time is considered explicitly—reduce carbon stocks more than a natural fire regime would have done (Mitchell et al. 2009). To formulate more generally, there is a cost associated with anticipation, which must not be ignored when evaluating risk management strategies (Seidl and Lexer 2013). Managing risks is therefore a balancing act between anticipating risks and fostering resilience, and a formal analysis of the predictability and available knowledge about the problem can help in finding a proper balance (figure 1).

A final property complicating the task of addressing uncertainty and risk in ecosystem management is their often paradoxical nature. Although I mentioned above an example of a risk-mitigation measure that can, itself, turn into a risk in another context, the opposite is also possible; that is, what constitutes a risk for one ecosystem service (or in one particular context) might be an opportunity for another service (or in another context). This can be illustrated aptly for natural disturbances: They present a major disruption of management and frequently lead to losses of a wide variety of ecosystem services. There is therefore a large interest in reducing the frequency and severity of disturbance events via anticipatory risk management. However, disturbances are also natural processes shaping ecosystems, they create a diversity of niches in the landscape and many species have coevolved with disturbance regimes over time. They therefore ultimately contribute to the resilience of ecosystems and are an integral part of managing for ecological diversity and integrity. In order to address risk factors such as natural disturbances in ecosystem management, we therefore need to overcome the fallacy that the source of risk and the source of safety are separated (Wildavsky 1988). Resolving this paradoxical nature of risk and putting it to use for ecosystem management remains a key challenge for future research (Holling and Meffe 1996). The lesson that we can already learn from this paradox, however, is that risk and uncertainty not only pose problems for the way we manage and use our natural resources, but they are, at the same time, opportunities to rethink and reinvent our interaction with the biosphere.

## Supplementary Material

Supplemental material

## Supplemental material

The supplemental material is available online at http://­bioscience.oxfordjournals.org/lookup/suppl/doi:10.1093/biosci/biu172/-/DC1.
