# Supplementary material for: The Shape of Ecosystem Management to Come: Anticipating Risks and Fostering Resilience
Source: Bioscience. 2014 Nov 5;64(12):1159–69. doi: 10.1093/biosci/biu172 (PMC4340566; doi:10.1093/biosci/biu172)
Supplement: Supplemental material [file supp_64_12_1159__index.html]

Supplemental material 

# The Shape of Ecosystem Management to Come: Anticipating Risks and Fostering Resilience

## Supplemental material

**Files in this Data Supplement:**

- Supplemental material
