# Supplementary material for: The Shape of Ecosystem Management to Come: Anticipating Risks and Fostering Resilience
Source: Bioscience. 2014 Nov 5;64(12):1159–69. doi: 10.1093/biosci/biu172 (PMC4340566; doi:10.1093/biosci/biu172)
Supplement: Supplemental material [file supp_biu172_Supplement_data.docx]

**Supplementary material**

***Risk and resilience in the ecosystem management literature***

I conducted a literature review of the peer-reviewed publications on risk and resilience in ecosystem management, using the Scopus database ([www.scopus.com](http://www.scopus.com)). The date of the query was July 25^th^ 2013, and besides the term “ecosystem management” the search terms risk and resilience where queried first separately and then in combination to determine the overlap between the two concepts in the literature (cf. Table S1). The results of the literature review show that research on risk accounts for approximately 7% of the current (2010-2012) literature on ecosystem management (Table S1). Research on resilience has quickly reached the same levels as risk research, with resilience issues being addressed in approximately 7% of the current literature on ecosystem management. And while the output of risk research has increased at approximately the same pace as the general ecosystem management literature (+7% yr^-1^ since the seminal report on the scientific basis of ecosystem management by Christensen et al. (1996)), research on resilience has increased at an even higher rate of +11% yr^-1^ (Figure S1).


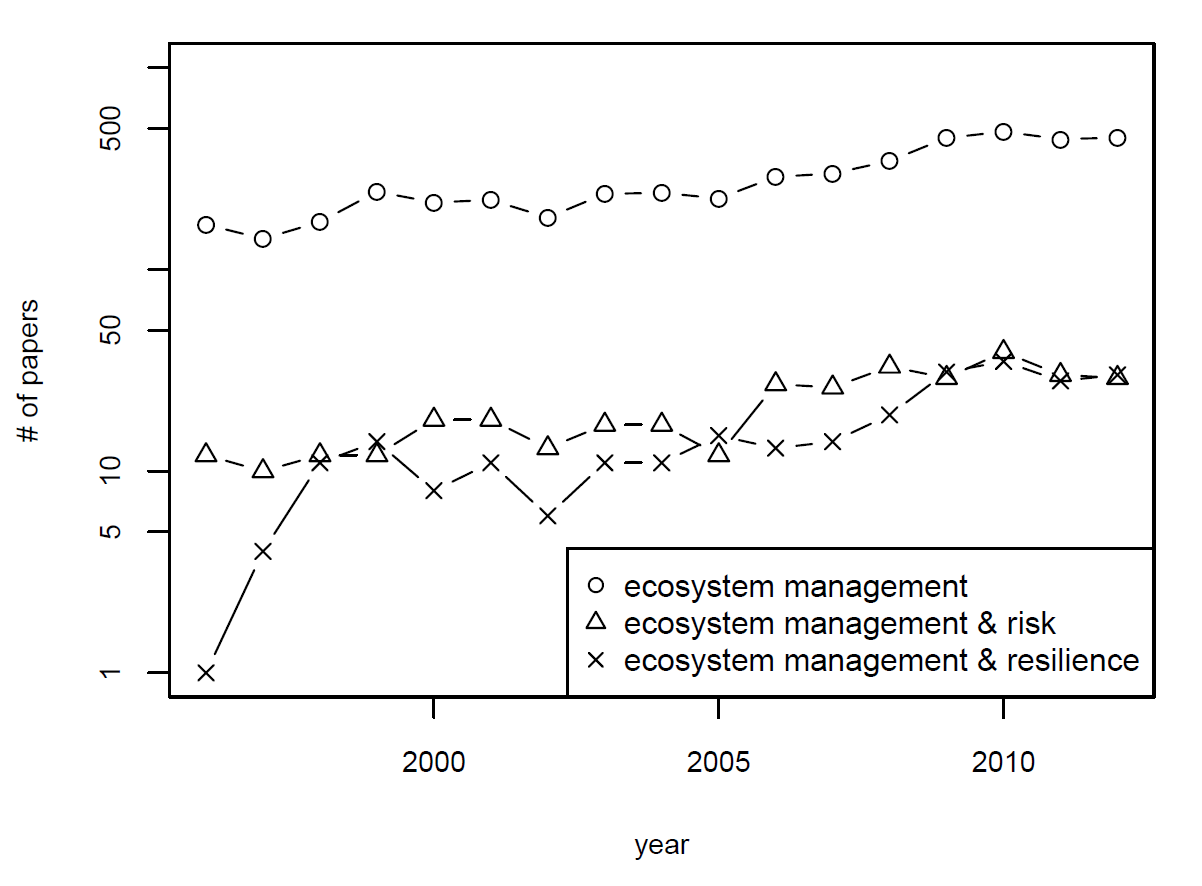


Figure S1: Papers published in the scientific literature addressing ecosystem management in general as well as risk, and resilience in ecosystem management in particular. Please note that the y axis is logarithmically scaled. For more details see Table S1.

Table S1: Literature published on risk and resilience in ecosystem management in the last 17 years. The Scopus database (www.scopus.com) was used to perform the literature search. Title, abstract, and key words were queried for the respective search terms (column headings), considering only the subset of papers including the term “ecosystem management”.

| year of publication | risk | | resilience | | risk & resilience | |
| --- | --- | --- | --- | --- | --- | --- |
|  | number of papers | % of the literature on ecosystem management | number of papers | % of the literature on ecosystem management | number of papers | % of the literature on ecosystem management |
| 1996 | 12 | 7.2 | 1 | 0.6 | 0 | 0.0 |
| 1997 | 10 | 7.0 | 4 | 2.8 | 0 | 0.0 |
| 1998 | 12 | 7.0 | 11 | 6.4 | 0 | 0.0 |
| 1999 | 12 | 4.9 | 14 | 5.8 | 0 | 0.0 |
| 2000 | 18 | 8.4 | 8 | 3.7 | 2 | 0.9 |
| 2001 | 18 | 8.1 | 11 | 5.0 | 1 | 0.5 |
| 2002 | 13 | 7.2 | 6 | 6.7 | 1 | 0.6 |
| 2003 | 17 | 7.2 | 11 | 4.6 | 1 | 0.4 |
| 2004 | 17 | 7.1 | 11 | 4.6 | 2 | 0.8 |
| 2005 | 12 | 5.3 | 15 | 6.7 | 1 | 0.4 |
| 2006 | 27 | 9.4 | 13 | 4.5 | 1 | 0.3 |
| 2007 | 26 | 8.7 | 14 | 4.7 | 1 | 0.3 |
| 2008 | 33 | 9.6 | 19 | 5.5 | 5 | 1.4 |
| 2009 | 29 | 6.5 | 31 | 6.9 | 3 | 0.7 |
| 2010 | 39 | 8.1 | 35 | 7.3 | 5 | 1.0 |
| 2011 | 30 | 6.8 | 28 | 6.3 | 5 | 1.1 |
| 2012 | 29 | 6.4 | 30 | 6.7 | 2 | 0.4 |

In total, issues of risk and resilience are addressed in more than 13% of the current works on ecosystem management, reflecting the growing importance of finding pathways to deal with an uncertain future in ecosystem services provisioning. However, only a small percentage (5%) of all works published on risk and resilience between 1996 and 2012 (that is 0.7% of the total corpus of the ecosystem management literature) address *both* aspects explicitly, while the overwhelming majority of studies exclusively uses terminology and approaches of either risk or resilience research. It has to be noted, however, that the peer-reviewed literature is only a weak proxy for describing the implementation of such concepts into practical ecosystem management, as several previous studies have found a considerable gap between science-based recommendations and their actual implementation, e.g., with regard to risk factors such as climate change (Blennow and Persson 2009, Kolström et al. 2011).

***Resolving trade-offs between anticipatory action and resilience-based measures***

In order to illustrate the application of the proposed framework I here include an example of tackling the risk of climate change in forest ecosystem management, particularly focusing on the question of how to achieve a meaningful balance between anticipation- and resilience-focused management. In order to tackle climate change in management planning, the Austrian Federal Forests (AFF) commissioned a research project on the climate change vulnerability of their ecosystem services provisioning (Seidl et al. 2011b). As with many other institutional strategies addressing risk, the starting point of the assessment was anticipatory in nature, using modeling-based scenario analyses to gauge climate risks, and working to prevent the thus expected negative impacts through implementing adaptation measures (Seidl et al. 2011a). Assessment indicators were selected and weighted in a stakeholder process, and included aspects of timber production, carbon storage, biodiversity, and disturbance. Indicators were combined by means of a multi-criteria analysis approach and a vulnerability surface (Luers 2005) was used to assess the overall effect of climate change on ecosystem services provisioning, as well as the efficiency of the evaluated adaptation measures (Seidl et al. 2011a, 2011b).

As an important subsequent step the robustness of this anticipatory strategy to uncertainties was evaluated, accounting for different climatic as well as societal scenarios (Seidl and Lexer 2013). This second analysis phase thus scrutinized the predictability of the problem (cf. the y axis in Figure 1), with increasing (between scenario) variation in ecosystem services provisioning indicating decreasing predictability. Using a standardized vulnerability index aggregating the different indicators of ecosystem service provisioning, low and medium predictability was found for 6.3% and 85.9% of the assessed management units, respectively (see Table S2). To elucidate the amount of knowledge available (cf. the x axis in Figure 1), an expert based management strategy designed to reduce the risks from climate change (Seidl et al. 2011a) was compared to the current business as usual management of the AFF (Seidl et al. 2011b). This analysis revealed that in 40.4% of the management units the expert based adaptation strategy did not improve ecosystem services provisioning under climate change over a continuation of business as usual management, indicating a lack of knowledge of how to address the expected climate change impacts. Overall, the signal of anticipatory adaptation clearly exceeded the noise of limited predictability in 35.5% of the assessed management units. On the other hand, for 41.6% of the study entities predictability and knowledge were low, suggesting that resilience should be the guiding principle in adapting them to climate change (Table S2).

It has to be noted that while such a quantitative uncertainty assessment can shed light on where anticipatory strategies are robust, they only consider a limited number of sources of uncertainty, and thus likely commit a type II error in favor of anticipatory measures. In order to address this problem, Seidl and Lexer (2013) did not only use management performance with regard to ecosystem service indicators as assessment criteria (as done here for illustrative purposes) but also explicitly included the adaptive capacity of the system in their assessment. This allowed for a simultaneous monitoring of how much an anticipatory measure reduces the potential to deal with unknown unknowns in the future, and thus safeguards against the fallacy of command-and-control management (Holling and Meffe 1996).

Table S2: Management units of the Austrian Federal Forests (%) assessed with regard to knowledge and predictability of ecosystem services provisioning under climate change (n=79, representing 129,000 ha of managed forest ecosystems). Five indicators of ecosystem services provisioning (i.e., productivity, timber stocks, carbon storage, biodiversity, and disturbance) were aggregated to a standardized vulnerability index [-1,1] by means of stakeholder-derived preference weights and thresholds. Predictability was assessed as the 95% confidence interval (*CI*) between the simulation-based estimates for different future scenarios of climate and societal preference. Knowledge was evaluated by comparing how much an expert-based adaptation strategy improved indicator performance compared to the current business-as-usual management ($\bar{M}$, averaged over all scenarios). The fields indicated in grey are the management units for which the effect of anticipatory adaptation is significant, i.e. where the signal of adaptation exceeds the noise of scenario uncertainty. For details on the methods and materials see (Seidl et al. 2011a, 2011b) as well as Seidl and Lexer (2013).

|  |  |  | knowledge of what to do | | |
| --- | --- | --- | --- | --- | --- |
|  |  |  | low  $\bar{M}$ ≤ 0.15 | medium  0.15 < $\bar{M}$ ≤ 0.30 | high  $\bar{M}$ > 0.30 |
| predictability of risk | high | $CI$ ≤ 0.15 | 1.3 | 1.3 | 5.1 |
|  | medium | 0.15 < $CI$ ≤ 0.30 | 36.6 | 20.2 | 29.1 |
|  | low | $CI$ > 0.30 | 2.5 | 2.5 | 1.3 |

***References***

Blennow K, Persson J. 2009. Climate change: Motivation for taking measure to adapt. Global Environmental Change 19:100–104.

Christensen NL, Bartuska AM, Brown JH, Carpenter S, D’Antonio C, Francis R, Franklin JF, MacMahon JA, Noss RF, Parsons DJ, Peterson CH, Turner MG, Woodmansee RG. 1996. The report of the Ecological Society of America committee on the scientific basis for ecosystem management. Ecological Applications 6:665–691.

Holling CS, Meffe GK. 1996. Command and control and the pathology of natural resource management. Conservation Biology 10:328–337.

Kolström M, Lindner M, Vilén T, Maroschek M, Seidl R, Lexer MJ, Netherer S, Kremer A, Delzon S, Barbati A, Marchetti M, Corona P. 2011. Reviewing the science and implementation of climate change adaptation measures in European forestry. Forests 2:961–982.

Luers AL. 2005. The surface of vulnerability: An analytical framework for examining environmental change. Global Environmental Change 15:214–223.

Seidl R, Lexer MJ. 2013. Forest management under climatic and social uncertainty: trade-offs between reducing climate change impacts and fostering adaptive capacity. Journal of Environmental Management 114:461–469.

Seidl R, Rammer W, Lexer MJ. 2011a. Adaptation options to reduce climate change vulnerability of sustainable forest management in the Austrian Alps. Canadian Journal of Forest Research 41:694–706.

Seidl R, Rammer W, Lexer MJ. 2011b. Climate change vulnerability of sustainable forest management in the Eastern Alps. Climatic Change 106:225–254.
